# White Matter Integrity of the Corpus Callosum and Psychopathological Dimensions in Deficit and Non-Deficit Schizophrenia Patients

**DOI:** 10.3390/jcm10112225

**Published:** 2021-05-21

**Authors:** Piotr Podwalski, Ernest Tyburski, Krzysztof Szczygieł, Katarzyna Waszczuk, Katarzyna Rek-Owodziń, Monika Mak, Piotr Plichta, Maksymilian Bielecki, Krzysztof Rudkowski, Jolanta Kucharska-Mazur, Wojciech Andrusewicz, Błażej Misiak, Agata Szulc, Anna Michalczyk, Sylwia Michałowska, Leszek Sagan, Jerzy Samochowiec

**Affiliations:** 1 Department of Psychiatry, Pomeranian Medical University, 71-460 Szczecin, Poland; kf.szczygiel@gmail.com (K.S.); zurawska1989@gmail.com (K.W.); krudkowski@gmail.com (K.R.); jola_kucharska@tlen.pl (J.K.-M.); annakarolina6@wp.pl (A.M.); samoj@pum.edu.pl (J.S.); 2 Institute of Psychology, SWPS University of Social Sciences and Humanities, 61-719 Poznan, Poland; etyburski@swps.edu.pl; 3 Department of Health Psychology, Pomeranian Medical University, 71-460 Szczecin, Poland; traasia@gmail.com (K.R.-O.); monika.mak@gmail.com (M.M.); piotrpp119@gmail.com (P.P.); maksbiel@gmail.com (M.B.); 4 Department of Neurosurgery, Pomeranian Medical University, 71-252 Szczecin, Poland; wojciech.andrusewicz@gmail.com (W.A.); leszekm.sagan@gmail.com (L.S.); 5 Department of Genetics, Wroclaw Medical University, 50-368 Wroclaw, Poland; mblazej@interia.eu; 6 Department of Psychiatry, Faculty of Health Sciences, Medical University in Warsaw, 05-802 Warsaw, Poland; agataszulc@poczta.onet.pl; 7 Department of Clinical Psychology, Institute of Psychology, University of Szczecin, 71-004 Szczecin, Poland; sylwiimichalowskiej@gmail.com

**Keywords:** corpus callosum, psychopathology, deficit schizophrenia, schizophrenia, diffusion tensor imaging, white matter integrity, fractional anisotropy, mean diffusivity

## Abstract

Deficit syndrome (DS) is a subtype of schizophrenia characterized by primary persistent negative symptoms. The corpus callosum (CC) appears to be related to psychopathology in schizophrenia. This study assessed white matter integrity in the CC using diffusion tensor imaging (DTI) in deficit and non-deficit schizophrenia (NDS) patients. We also investigated the psychopathological dimensions of schizophrenia and their relationship to CC integrity. Fifteen DS patients, 40 NDS patients, and 30 healthy controls (HC) underwent psychiatric evaluation and neuroimaging. We divided the CC into five regions and assessed their fractional anisotropy (FA) and mean diffusivity (MD). Psychopathology was assessed with the Positive and Negative Syndrome Scale. DS patients had lower FA than NDS patients and HC, and higher MD in Region 5 of the CC than did HC. NDS patients had higher MD in Region 4 of the CC. The patient groups differed in terms of negative symptoms. After differentiating clinical groups and HC, no significant correlations were observed between DTI measures and psychopathological symptoms. Our results suggest that DS and NDS are characterized by minor impairments of the posterior CC. We confirmed that DS patients have greater negative psychopathology than NDS patients. Our results are preliminary, and further studies are needed.

## 1. Introduction

Schizophrenia is a chronic disease with a complex etiopathogenesis and poor prognosis. It is more common in men (the ratio of men to women who suffer from this disease is 1.4:1), and its median lifetime risk is estimated at 7.2 in 1000 people [1]. Schizophrenia is characterized by disturbances in perception, thinking, emotions, and cognitive functions, and it results in a wide variety of difficulties in everyday functioning [2,3,4]. It is a very heterogeneous disease; it may be possible to distinguish between different subtypes of this disease using biological markers [5]. One of the subtypes of this disease is deficit syndrome (DS), which was first described by Carpenter in 1998 [6]. This subtype is characterized by dominant and persistent negative symptoms, such as poverty of speech, social withdrawal, apathy, and blunt affect. These symptoms are primary, meaning that they are not caused by positive symptoms of the disease, neuroleptic treatment, or other medical conditions, and that they persist over time [7,8]. A better understanding of DS is extremely important due to its poorer prognosis [9].

As schizophrenia has a complex psychopathology, the identification of subtypes of schizophrenia can help organize its complex clinical outline; however, it is also important to understand the characteristics of the symptoms, and attempts have been made to do so. Much effort has been expended in describing its heterogeneous symptomatology [10]. Since Kraepelin’s time, one significant change in the conceptualization of the disease has been the division of its symptoms into two dimensions: positive and negative [11]. Positive symptoms include delusions or hallucinations, whereas negative deficit symptoms include blunted affect, alogia, asociality, anhedonia, and avolition [12]. The creation of psychometric scales has made possible the measuring of these dimensions. The Positive and Negative Syndrome Scale (PANSS) consists of 30 items grouped into three dimensions: positive (seven items), negative (seven items), and general psychopathology (16 items) [13]. On the basis of this scale, further psychopathological factors have been distinguished, and they are extremely useful for assessing the disease process in clinical and scientific contexts. These factors are: positive, negative, disorganized, affect, and resistance. The five, six, or seven factor PANSS model seems to be an adequate and complete description of the disease [14].

The neurodevelopmental hypothesis is one attempt to explain the etiopathogenesis of schizophrenia. It suggests that a genetic basis interacts with environmental factors at some point in an individual’s life, leading to disturbances in the structures of the central nervous system and thus to psychopathological symptoms [15]. There is also evidence that immune dysregulation and neuroinflammation play a role in the pathogenesis of schizophrenia. This is confirmed, inter alia, by reports of altered concentrations of various inflammatory cytokines in schizophrenia patients [16]. It can lead to structural and functional disorders in white matter (WM), which is responsible for communication between different areas of the brain [17].

The etiology of schizophrenia has often been hypothesized as being related to disconnection. One of the first theories of disconnection was proposed by Wernicke. It is now believed that the underlying cause of schizophrenia is disturbances in connections between functional areas of the brain and not, as originally assumed, of single connections between specific neuroanatomical areas [18]. These disturbances would be due to changes in the properties of WM bundles. It is believed that, in schizophrenia, the most important disruptions of interactions may occur in the prefrontal cortex, the temporal cortex, and the limbic system [19]. WM is part of the nervous tissue and mainly consists of the axonal parts of neurons and oligodendrocytes. Histopathological research has confirmed the existence of disturbances in the structure of myelin and oligodendrocytes in schizophrenia [20]. 

Diffusion tensor imaging (DTI) is an imaging method that enables the assessment of the properties of water diffusion in tissues. In highly ordered tissues (e.g., WM), water diffuses in a more ordered (anisotropic) manner compared to less ordered tissues (e.g., grey matter). The measures of fractional anisotropy (FA) and mean diffusivity (MD) allow us to assess the diffusion properties of a tissue. FA values range from 0 to 1, where 0 indicates isotropic diffusion (completely disordered) and 1 indicates completely anisotropic diffusion. MD is an indicator of overall tissue diffusivity. Low values of this parameter indicate that a structure is highly ordered. Thus, DTI enables the in vivo assessment of the microstructure of WM [21]. WM integrity studies in patients with schizophrenia have shown significantly reduced FA values in many WM structures [22].

The largest WM structure in the human brain is the corpus callosum (CC) at the bottom of the cerebral fissure. It is the main interhemispheric commissure. The CC begins to form around day 74 of gestation, ending around day 115, proceeding from the anterior towards the posterior [23]. The CC plays the main role in integrating information from both hemispheres; it controls thought as well as emotional and behavioral processes [24]. The participation of the CC in the pathogenesis of psychosis is indicated by reports of cases of psychotic symptoms when partial or complete agenesis of the CC has occurred [25].

The CC plays a major role in the integration of higher mental functions, including cognitive functions, language organization, and executive functions [26]. Thanks to the development of magnetic resonance imaging (MRI), there is evidence of decreased CC volume in schizophrenia [27]. These changes seem to be already present during the first episode of psychosis [28]. However, such changes have not been detected in childhood-onset schizophrenia [29]. By using DTI, it is possible to study the integrity of the CC microstructure. Recent DTI studies predominantly indicate a reduction of FA in major WM bundles, including the CC. These changes may have an important role in the pathophysiology of this psychiatric condition. They indicate a disturbance in the interhemispheric communication of different cortical areas [22,30,31]. Changes in the integrity of this structure are already observed in people experiencing their first psychotic episode [32], which may indicate that it is important in the pathogenesis of schizophrenia. Moreover, these disturbances may be correlated with the duration of the disease [33]. Madigand et al. compared macrostructural and microstructural changes in patients with short and long courses of the disease and found differences in the distributions of volume changes in the CC. The reduction in CC volume was notable in the anterior part of the bundle in the long course of schizophrenia, in contrast to the microstructural changes that were already observed in both groups. Therefore, it seems that integration (both macro- and micro-structural) of the CC can be an indicator of the duration of the illness. Impairment of integrity (reduced FA) progresses gradually from the posterior to the anterior of the CC [33]. However, del Re et al. found reduced volume in the central part of the CC [34]

To date, only a few studies have assessed WM structure in DS patients. They found a reduction in FA values in various brain regions in patients with DS compared to those with non-deficit schizophrenia (NDS). This may indicate increased WM disruption in the DS population [35,36,37,38,39,40]. Voineskos et al. [37] found that in patients with first-episode psychosis with clinical correlates of DS, there is greater disruption of WM tracts. This provides evidence that clinical findings of DS may be derived from such disruptions. Disruption of WM in the superior longitudinal fasciculus and uncinate fasciculus points towards the hypothesis of disconnectivity between the temporal, parietal, and limbic cortices [35,36]. Only some of these studies examined the CC [37,38,39]. Reduction of FA in the CC was described by Spalletta et al. [40] and by Lei et al. in a larger sample [38]. This suggests that not only frontotemporal miscommunication, but also communication between posterior parts of the brain and interhemispheric is crucial to understanding the psychopathology of DS. There is also disagreement as to the area in which integrity disorders within this bundle occur. Reduced FA in the splenium [37] and the body of the CC [39] has also been described. As previous results are inconsistent and because the location of the WM impairment can have psychopathological consequences (because of interhemispheric cortical projections), we decided to focus on identifying whether disruptions in specific regions of the CC may be involved in the pathophysiology of DS.

The literature also suggests that the integrity of the CC may be related to different clinical factors and psychopathological symptoms [22]. DTI studies have established a relationship between psychopathology and changes in diffusion parameters within the CC in patients with schizophrenia [41]. Already in the first psychotic episode, there are changes in the CC which correlate with the intensity of positive symptoms [32]. Abnormal asymmetry of the integrity of the CC projection fibers, especially in the anterior part of the frontal area and in the posterior part of the occipital area, may be related to cognitive impairment [42]. Studies have emphasized the relationship between increased radial diffusivity within the CC and social indices, especially the Picture Arrangement subtest of the Wechsler Adult Intelligence Scale [43]. The relationship between drug resistance and CC integrity is also interesting. One study that compared treatment-resistant schizophrenia (TRS) patients with patients with non-treatment-resistant schizophrenia showed a considerable reduction in FA in the splenium of the CC in TRS patients; the authors suggested that changes in the CC may be a marker of treatment-resistant schizophrenia [44]. 

Research on the CC may prove important for determining the neurobiological background of schizophrenia. This biological background may vary between different patient populations. It seems that changes in the callosal fibers have the potential to help differentiate these groups. Identification of homogeneous groups in schizophrenia and of differences between individual populations may lead to the identification of specific biomarkers for individual subgroups [45]. The search for such biomarkers, which would function as tools with high sensitivity and specificity, is an important current challenge for psychiatry. As diagnosis is still a process based on subjective-descriptive classification, objective ways of making diagnoses are needed. In addition to diagnosis, biomarkers can also measure specific parameters, control treatment outcomes, and help predict psychopathological factors. Neuroimaging with DTI exploration is one of the focal points of research into biomarkers [5].

As mentioned above, to date there have been few studies on patients with the deficit subtype of schizophrenia that have used DTI. To the best of our knowledge, specific regions in the CC and their relationship with psychopathological dimensions have not yet been investigated in this group. We hypothesize that there is a difference between DS, NDS, and healthy controls (HC) in WM integrity in the CC. Our second hypothesis is that DS patients differ from NDS patients in psychopathological dimensions. We also hypothesize that there is a relationship between changes in the integrity of the CC and the psychopathology of DS. In light of this, the first aim of this study was to compare WM integrity in the CC using DTI in DS patients, NDS patients, and HC. The second aim was to compare the severity of different psychopathological dimensions between both clinical groups. If significant differences in DTI are found between clinical groups and HC, then the third aim will be to examine the relationship between the integrity of the largest WM bundle and psychopathology in DS and NDS patients. We have implemented this approach based on the propositions of Harms et al. and Whitford et al. [46,47]. The essence of this study will be the assessment of specific areas of WM using the CC topographical segmentation method proposed by Hofer et al. [48]. The results of this study may be important for the identification of biomarkers of schizophrenia.

## 2. Materials and Methods

### 2.1. Participants

A total of 55 participants were recruited from among patients under the care of the Department of Psychiatry of the Pomeranian Medical University in Szczecin, Poland. A large proportion were inpatients, but some were recruited from day wards and the outpatient clinic. Participants were recruited on the basis of a diagnosis of schizophrenia in accordance with the diagnostic criteria from the International Statistical Classification of Diseases and Related Health Problems ICD-10 [49]. A structured clinical interview for DSM-IV and ICD-10 (the Mini-International Neuropsychiatric Interview; MINI) was used for this [50]. The inclusion criteria for the study included the patient’s age (between 30 and 55 years), having been diagnosed with schizophrenia for at least ten years, the ability to undergo a psychiatric examination and a neuroimaging procedure, the ability to provide informed consent, and a stable mental state. The exclusion criteria were substance use disorders, a history of cerebral or cranial injuries, severe somatic conditions, and psychiatric or neurological comorbidities. All patients gave written consent to participate in the study. The study protocol was approved by the local bioethics committee.

The control group consisted of 30 healthy participants matched to the examined group of subjects in terms of sex, age, and years of education. It was composed of healthy people without psychiatric, neurological, or severe somatic diseases, as confirmed by precise medical examination and a structured, self-constructed clinical interview containing a number of questions about the state of their health. The exclusion criteria were the same as for the study group, apart from the diagnosis of schizophrenia. None of the control participants were excluded on this basis. Participation in the study was voluntary and all participants gave written informed consent.

### 2.2. Clinical Assessments

The evaluation of the diagnosis started with a structured interview using the MINI. This enabled a reliable diagnosis and the exclusion of psychiatric comorbidities. Then, the patients were examined using the Positive and Negative Syndrome Scale (PANSS) [13], which assessed the psychopathological functioning of the patients. We distinguished the following dimensions: positive, negative, disorganized, affect, and resistance [14]. The diagnosis of DS was made after a psychiatric examination based on the criteria proposed by Kirkpatrick adopted for the ICD-10 [51] (see Table 1); these concern the primary symptoms characteristic of this syndrome, such as social withdrawal, blunted affect, apathy, speech poverty, and anhedonia. These symptoms could not be due to pharmacotherapy, psychotic state, or other medical reasons. To ensure an accurate diagnosis, we supplemented this with a proxy for the deficit syndrome—the PANSS, a tool with agreed, adequate, and stable psychometric properties [37,51,52]. The Polish versions of the Brief Negative Symptom Scale (BNSS) [12] and the Self-evaluation of Negative Symptoms (SNS) [53] were used to describe the symptoms of DS. The functioning of all patients was also assessed using the Global Assessment of Functioning (GAF) [54]. All patients who participated in the study were treated with neuroleptics in accordance with good clinical practice and recommendations. None of the patients were in acute psychosis.

### 2.3. Image Acquisition

DTI data were acquired using a 3.0 Tesla scanner (General Electric Signa HDxt, Milwaukee, WI, USA). In this procedure, we used a single shot pulse sequence. Imaging parameters were: diffusion-weighted, echo planar acquisition; TR = 11,675 s; TE = 82.80 ms; numbers of excitation (NEX) = 2; matrix = 96 × 96; field of view = 240 mm × 240 mm; slice thickness = 3 mm; slice gap = 0.50; acquisition time = 10 min, 19 s. Diffusion images were obtained along 25 gradient directions (b value = 1000 s/mm^2^).

### 2.4. Image Processing and Quality

**Figure 1:**
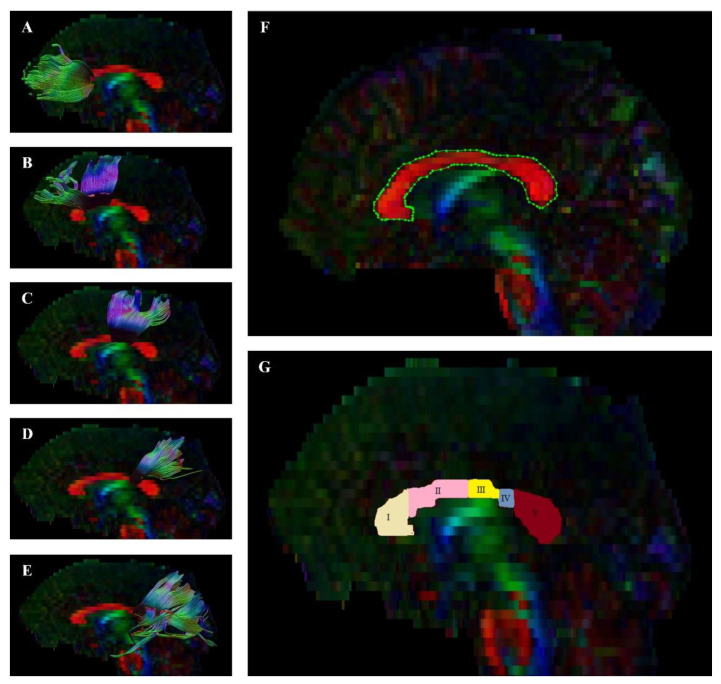
Diffusion tensor tractography of the corpus callosum (CC) with fractional anisotropy (FA) color maps. Green, red, and blue colors represent fibers running along the axis (anterior–posterior, left–right, and superior–inferior, respectively). Five single projections with ROIs on a sagittal plane ((**A**) = Region 1, (**B**) = Region 2, (**C**) = Region 3, (**D**) = Region 4, and (**E**) = Region 5), and (**F**) = the whole region of the CC, (**G**) = all regions of the CC.

We performed preprocessing, quality control, and fiber tract visualization with the ExploreDTI tool [55]. We first converted DICOM files to the *.nii format, which is compatible with this program. We then checked whether the sides of the converted images matched the originals. Next, we corrected data for signal drift, removed artifacts (such as Gibbs ringing), and corrected effects due to motion and eddy current. Based on this data, we performed whole-brain tractography. To visualize the whole CC, we used a single region of interest (ROI) on the sagittal plane. Then, we divided the CC into 5 single ROIs on the sagittal plane using the method proposed by Hofer et al. [48] (Figure 1). This segmentation is mainly based on connections of callosal fibers projecting to the cortical regions in each hemisphere. It allows us to distinguish five specific regions: Region I sends its projections to the prefrontal cortex; Region II sends its projections to the premotor and supplementary motor cortices; Region III sends its projections to the motor cortex; Region IV sends its projections to the sensory cortex; and, finally, Region V sends its projections to the parietal, temporal, and occipital cortices. Following this, we excluded parts of tracts that were not anatomically involved with the “ROInot” regions. Fractional anisotropy of the fiber tract was calculated automatically by the ExploreDTI Descriptive Statistics function.

### 2.5. Procedure

The entire procedure always took place within one day. It usually took place in the middle of the week between 8 a.m. and 3 p.m. The first element was the psychiatric examination that was required in each case. A trained psychiatrist evaluated the subject using tools with confirmed psychometric properties (PANSS, MINI, GAF, BNSS, SNS). All patients then underwent the imaging protocol.

### 2.6. Statistical Analysis

Statistical analysis of the results was done using IBM SPSS 26 (IBM Corp., Redmont, VA, USA). Continuous variables were presented as means (*M*) and standard deviations (*SD*). The normalities of the distributions were examined with the Shapiro–Wilk test as well as the skewness and kurtosis values. We assumed that skewness between −2 to +2 and kurtosis between −7 to +7 indicated normal distribution of variables [56]. Age and all DTI parameters (FA and MD) were normally distributed in all three groups, chlorpromazine equivalent and global functioning on the GAF were normally distributed in both clinical groups, and psychopathological dimensions were normally distributed only in the DS group. Differences between two groups were examined with Student’s *t* test (if the relevant assumptions were met) and the Mann–Whitney *U* test (if the relevant assumptions were not met). Differences between three groups were examined with the one way analysis of variance (ANOVA) *F* test (if the relevant assumptions were met) and the Kruskal–Wallis *H* test (if the relevant assumptions were not met). The Bonferroni post hoc test was used for comparisons between groups (for parametric tests, as there were no group differences after using the non-parametric *H* test). Cohen’s *d* and the η^2^ effect size (parametric tests) [57] or Wendt’s *r*_U_ and *E* (non-parametric tests) [58] were used to determine the magnitudes of effect sizes for all statistical tests. In cases where there were significant differences in DTI measures between both clinical groups, analysis of covariance (ANCOVA) was performed to control the effect of negative symptoms, because this variable might be related to WM integrity [59]. Finally, in order to assess the relationship between the DTI measures and psychopathological symptoms in both clinical groups, Pearson’s *r* and Spearman’s *rho* correlation coefficients were estimated, respectively. Holm–Bonferroni *p*-value correction was used for all statistical analyses (multiple comparisons and correlations). The alpha criterion level was set at 0.05 and a statistical power above 0.80 for all statistical analyses [57]. However, we considered values less than 0.1 to constitute trends (0.05 < *p* < 0.1), which is recommended in the literature for biomedical sciences [60].

## 3. Results

### 3.1. Characteristics of Participants

Demographic and clinical characteristics are presented in Table 2. There were no significant group differences in age, years of education, or sex. After Holm–Bonferroni *p*-value correction, the clinical groups did not differ significantly in antipsychotic medications, chlorpromazine equivalent, duration of illness, exacerbations, treatment interruptions, or global functioning as measured by the GAF.

### 3.2. Differences in DTI Measures

**Figure 2:**
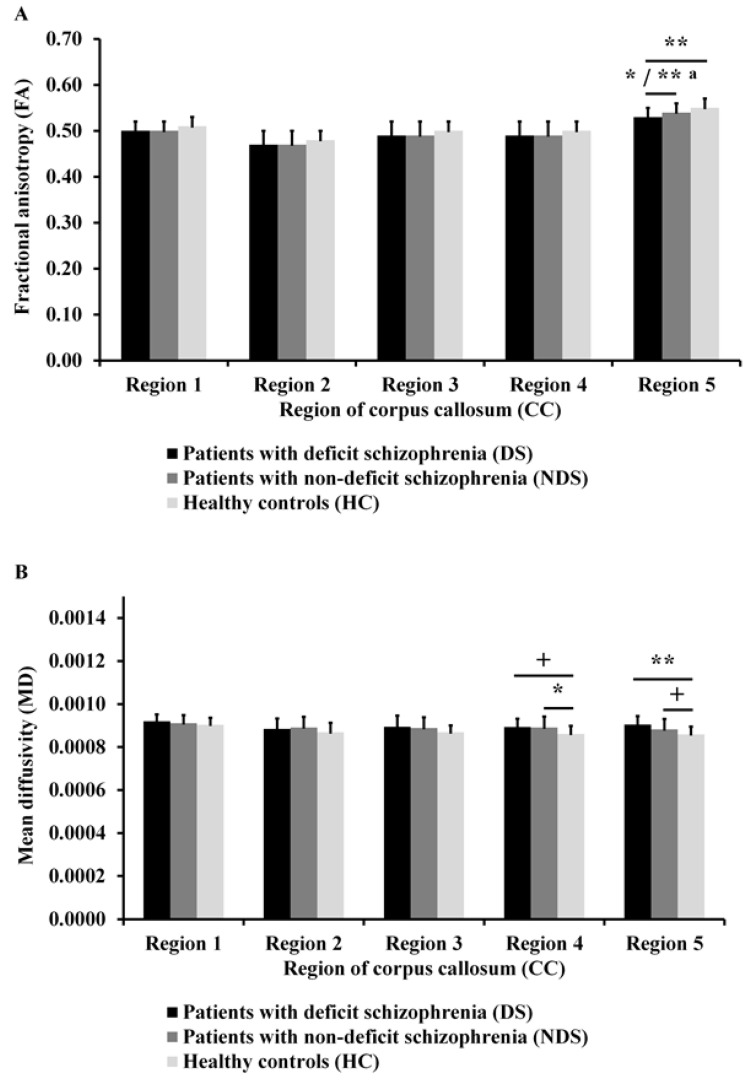
Fractional anisotropy (**A**) and mean diffusivity (**B**) of the corpus callosum (CC) for all groups. Standard deviations (*SD*) are presented as bars. ^a^ Significant difference after co-varying the negative symptoms; ^+^ *p* < 0.1; * *p* < 0.05; ** *p* < 0.01.

As can be seen in Figure 2A, there was a significant difference in FA in Region 5 of the CC (*F*_(2, 82)_ = 6.86; *p* = 0.002; η^2^ = 0.14; statistical power = 0.91) between the three groups. Post hoc analyses showed that DS patients had lower FA in Region 5 of the CC than NDS patients and HC (*p* = 0.027 and *p* = 0.001, respectively). Differences in this region between both clinical groups (*F*_(1, 52)_ = 9.72; *p* = 0.003; η^2^ = 0.16; statistical power = 0.86) remained significant after co-varying for negative symptoms.

As can be seen in Figure 2B, there were significant differences in MD in Region 4 (*F*_(2, 82)_ = 4.51; *p* = 0.014; η^2^ = 0.11; statistical power = 0.76) and Region 5 of the CC (*F*_(2, 82)_ = 6.03; *p* = 0.004; η^2^ = 0.12; statistical power = 0.87) between the three groups. Post hoc analyses showed that DS patients had higher MD than HC in Region 5 of the CC (*p* = 0.003) and in Region 4 of the CC (*p* = 0.070) on the level of trend toward statistical significance. However, NDS patients had higher MD than HC in Region 4 of the CC (*p* = 0.019) and in Region 5 of the CC (*p* = 0.089) on the level of trend toward statistical significance.

### 3.3. Differences in Psychopathological Dimensions

**Figure 3:**
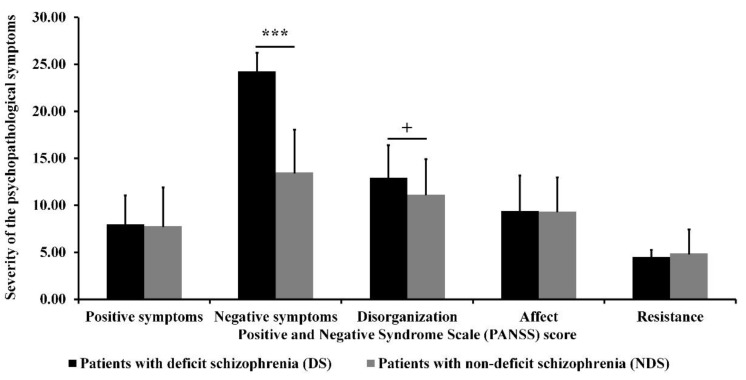
Severity of different dimensions of psychopathological symptoms for both clinical groups. Standard deviations (*SD*) are presented as bars. ^+^ *p* < 0.1; *** *p* < 0.001.

As can be seen in Figure 3, DS patients had greater severity of negative symptoms than NDS patients (*Z* = −5.17; *p* < 0.001; *r*_U_ = 0.94; statistical power = 1.00). After Holm–Bonferroni *p*-value correction, there were no significant differences between the clinical groups in the severity of the other four dimensions of psychopathological symptoms. However, the difference between groups in disorganized symptoms trended towards statistical significance (*Z* = −2.12; *p* = 0.034).

### 3.4. Relationship between DTI Measures and Psychopathological Dimensions

Table 3 shows the correlations of FA and MD in the CC (for which there were significant differences to HC) with different dimensions of psychopathological symptoms in both clinical groups separately after Holm–Bonferroni *p*-value correction. No statistically significant correlations were observed between DTI measures and psychopathological symptoms.

## 4. Discussion

This study analyzed five specific regions of the CC in DS and NDS patients. DTI analysis allowed us to reconstruct the CC projections and examine the diffusion parameters in all subjects. Using the segmentation of CC proposed by Hofer [48], we explored changes in CC integrity and their relationship with psychopathology in patients with DS and NDS (when there were differences between the clinical groups and HC).

Our first hypothesis was that there would be a difference between DS, NDS, and HC in WM integrity in the CC—the results partially confirmed this. Using DTI to assess diffusion parameters, we identified changes in WM integrity within this structure. We found that these changes were unevenly located across the CC. DS patients showed a reduction in FA values in the posterior CC region compared to NDS patients and HC. These changes were partially consistent with other observations in both patients with chronic and first episode schizophrenia [32,61]. Changes in WM integrity were consistent with other studies, showing a decrease in FA values in CC in the DS subpopulation [37,38,39,40]. Changes in the values of diffusion parameters may be related to reductions in fiber integrity, density of cell membranes, quality of myelinization, or glial cells [62]. Disturbances in the functionality of oligodendrocytes can cause changes in axon myelination and thus changes in diffusion parameters and, more importantly, probable disturbances of intracortical connections that may result in the symptomatology of schizophrenia. One of the reasons for this may be the neuroinflammatory process that is widely documented in schizophrenia. Activation of various immune system cells can lead to the destruction of glial tissue and thus changes in the integrity of WM. Neuropathological studies have shown a decreased number of astroglial cells in, inter alia, the anterior part of the CC, as well as increased transcription of pro-inflammatory cytokines in the area of the fronto-orbital cortex [17]. It has been reported that alterations in levels of proinflammatory cytokines could differ between DS and NDS patients. Elevated interleukin-6, interleukin-17, and C-reactive protein have been observed in DS patients [63,64]. Together with neuroimaging findings, this can help in the stratification of subtypes of schizophrenia. This is in line with the disconnection hypothesis, which agrees with the neurodevelopmental theory [16]. A link between MIR137 and reduction of FA has been observed in families with a high risk of psychosis. This gene is involved in neurodevelopment, and thus can link the connective and neurodevelopmental hypotheses [65]. However, the genetic aspects of schizophrenia require further exploration. 

Moreover, this study found a difference in MD in the CCs of DS patients compared to HC. Our data showed that patients with DS had greater MD in Regions 5 and 4 (only on the level of trend toward statistical significance) of the CC compared to HC. However, the NDS patients differed from the HC in Region 4 and 5 (again on the level of trend).

According to the CC scheme used here, Region 4 comprises projections to the cortical sensor regions. In contrast, Region 5 covers the posterior quarter of the CC and sends projections to the parietal, temporal, and occipital cortex regions. Regions 4 and 5 may anatomically correspond to the splenium of CC. Reduced FA has been found in this area in schizophrenia patients compared to controls [66]. The integrity disorders in this area documented in our study may cause changes in intracellular parietal–occipital connections. Structural and functional changes in the occipital area in schizophrenia have been described [67]. They may be related to the abnormalities in visual processing associated with schizophrenia, and interestingly, decreased FA in this region is related to impaired facial emotion recognition [68,69]. It seems that this may be related to the diminished affective and emotional reaction disorders observed in DS. Reduction of volume of gray matter in the parieto–occipital region is positively correlated with the severity of negative symptoms [70]. It is worth noting that one study assessed the functioning of WM, including in the CC, and the impairment of empathy in patients suffering from schizophrenia and found a negative correlation between the personal distress subscale of the Interpersonal Reactivity Index (which assesses “self-oriented” feelings of personal anxiety) and FA in the splenium of the CC [71].

Our second hypothesis posited differences in psychopathological dimensions between DS and NDS patients; the results confirmed the existence of such differences. We were able to observe differences in psychopathology within the negative dimension of the disease spectrum. Patients characterized as DS in our study scored higher on the following items: blunted affect, emotional withdrawal, poor rapport, passive apathetic social withdrawal, lack of spontaneity, motor retardation, and active social avoidance (i.e., the negative items according to Shafer et al. [14]). This is in line with other studies on the psychopathological characteristics of DS patients [72,73,74]. The participants in our group did not differ significantly in terms of age, gender, or in the number of psychotic exacerbations or interruptions to treatment. The groups did not differ in levels of general functioning as measured by the GAF scale. This is inconsistent with other reports [73] and may be due to our study group being of insufficient size. We observed the prevalence of disorganization symptoms in DS patients, but this turned out to be insignificant after Holm–Bonferroni correction. The meta-analysis of Cohen et al. [75] reported much more severe negative symptoms and slightly more severe disorganization symptoms in DS patients. The lack of differences in psychopathology in other dimensions is probably due to study design. All participants were not in the acute state of psychosis. This may also indicate that the negative symptoms were not due to another psychopathology, but were primarily according to the DS criteria.

The results did not confirm the third hypothesis, which concerned a link between changes in the integrity of the CC and the psychopathology of DS. This study did not find any relationship between changes in diffusion values (when there were differences between the clinical groups and HC) and psychopathology in either clinical group. This also may be related to the size of the groups. Our results are in line with previous findings [76,77,78], but not with those from the study by Ohoshi et al. [59], who found an association between FA in the parietal parts of the CC with positive, negative, and general psychopathology in schizophrenia. Our approach was based on Harms et al. [46] and Whitford et al. [47], who examined correlations between DTI measures and psychopathological symptoms when there were significant differences in DTI between patients with schizophrenia and HC. The abnormality in connections can be produced by impairment in conductivity and desynchronization of axonal communication. It seems that the diffusion parameters obtained in DTI may be involved in that process. Likewise, it appears that this pathomechanism could be necessary for the onset of psychopathology. This approach seems to be justified: if there are changes in the structure of WM, it can be expected that they are related to the symptoms of the disease. Moreover, due to the fact that disease duration, pharmacological treatment, and social functioning have been shown to be associated with DTI measures and psychopathological symptoms [46,79,80], it is necessary to be very careful when interpreting the obtained results, because due to the small size of the study groups, we did not perform a complex regression analysis controlling for these confounding variables. Thus, more research is needed on this topic.

Direct linking of diffusion parameters obtained with the use of DTI techniques is generally very difficult. The results concerning the relationship of the CC with the psychopathology of schizophrenia are inconclusive. In male patients suffering from schizophrenia, there are reports of a positive correlation between FA values in the CC and positive symptoms and total score on the PANSS scale, as well as on the negative symptom scale [81]. However, after Holm–Bonferroni correction, these correlations became less strong [41,82]. In contrast, there are reports of negative correlations of FA in the CC with positive symptoms [83] and negative symptoms [59,76,83,84]. This ambiguity is exacerbated by reports that did not find any links between psychopathology and diffusion parameters [85]. This may be due to lack of stratification into subpopulations. Schizophrenia as a heterogeneous disease may, on one hand, present a diverse clinical picture and, on the other hand, perhaps have different neurobiological bases. Other causes of ambiguity in the correlations may be the sample sizes being too small, pharmacotherapy and treatment history, the duration of the disease, the duration of untreated disease, or the severity of symptoms and methods used to evaluate them.

In the population we studied, the duration of the disease in patients with DS was generally longer than in the NDS group. This result was statistically insignificant after Holm–Bonferroni correction for multiple comparisons. The duration of the disease affects the integrity of the WM within the CC. FA reduction in long-term patients is present in the posterior and anterior of the CC, in contrast to short-term patients where FA reduction was detected in the posterior part. Furthermore, macrostructure changes and reduction of CC volume were observed only in the population of long-term patients [33].

This study has some limitations. The first being that the studied groups were small, which limited the statistical analysis. It is important to emphasize that there are few studies on the deficit subtype using DTI, and most were conducted on a rather small number of subjects. Moreover, the cross-sectional design of the study itself prevents the identification of cause-and-effect relationships between changes in diffusion values and the deficit subtype. Longitudinal studies determining, for example, the relationship between the integrity of WM and the course of the disease may prove helpful here. Another limitation is the ambiguity in the interpretation of diffusion parameter values in the deficit subtype. It seems advisable to conduct post mortem neuropathological studies on this group of subjects. Furthermore, DTI imaging itself has limitations: for example, low resolution could lead to misinterpretation of the data. We believe that because of the importance of the CC in the functioning of the central nervous system, it would be beneficial to investigate this area in various populations of people with mental disorders and diseases. Perhaps such research would shed light on the potential common pathological basis of various psychiatric states, such as autism, psychotic disorders, affective disorders, and personality disorders [62]. Another limitation may be that the duration of untreated psychosis was not taken into account. This may affect the quality of diffusion within the nervous tissue in the central nervous system. Finally, the effect of neuroleptic treatment on WM integrity remains unknown. The participants in our study were being treated with different neuroleptics at different doses. Perhaps conducting detailed studies with specific medications in the future may enable us to better plan and control research and facilitate the planning of various therapies for patients with schizophrenia. Future research should also consider the use of different modalities of neuroimaging techniques. This would help explore the relationship between functional and structural changes in connections between different cortical areas, and, hence, to paint a clearer picture of the disconnection hypothesis.

## 5. Conclusions

This study to assess the structure of individual CC regions in deficit and nondeficit schizophrenia was based on the division proposed by Hofer et al. [48]. We found WM integrity disorders in the posterior part of the CC in patients suffering from schizophrenia. Subjects with DS may differ from NDS patients, with significantly greater changes in these areas. This corroborates the existence of differences at the structural level between DS and NDS patients. At the same time, we found that DS patients have more negative symptoms than NDS patients. This points towards the validity of this subtype of schizophrenia, as proposed by Carpenter et al. [6]. No correlation was found between changes in WM integrity (which were detected compared to HC) and psychopathological symptoms in DS and NDS patients.

## Figures and Tables

**Table 1:** Diagnostic criteria for deficit syndrome (DS) of schizophrenia (based on Kirkpatrick 1993, modified) [51].

At least two of the following six negative symptoms must be present:
a. Restricted affect
b. Diminished emotional range
c. Poverty of speech
d. Curbing of interests
e. Diminished sense of purpose
f. Diminished social drive
Some combination of two or more of the negative symptoms listed above has been present for the preceding 12 months and always was present during periods of clinical stability (including chronic psychotic states). These symptoms may or may not be detectable during transient episodes of acute psychotic disorganization or decompensation.
The negative symptoms above are primary (i.e., not secondary to factors other than the disease process). Such factors include:
a. Anxiety
b. Drug effect
c. Suspiciousness (and other psychotic symptoms)
d. Mental retardation
e. Depression
The patient meets ICD-10 criteria for schizophrenia.

**Table 2:** Demographic and clinical characteristics of all groups.

	Patients with Deficit Schizophrenia (DS) (*n* = 15)	Patients with Non-Deficit Schizophrenia (NDS) (*n* = 40)	Healthy Controls (HC) (*n* = 30)	*F*/*H*/χ^2^/*t*/*Z*
Age: *M* (*SD*)	39.80 (6.14)	38.83 (7.24)	37.30 (8.24)	0.66 ^a^
Years of education: *M* (*SD*)	12.80 (2.60)	13.40 (2.63)	14.53 (2.60)	4.85 ^b^
Sex: female/male	4/11	22/18	16/14	3.79 ^c^
Antipsychotic medications:				
Atypical: *n* (%)	11 (73.33)	26 (65.00)	-	1.24 ^c^
Atypical and typical: *n* (%)	4 (26.67)	11 (27.50)	-	
Typical: *n* (%)	0 (0.00)	2 (5.00)	-	
No medications: *n* (%)	0 (0.00)	1 (2.50)	-	
Chlorpromazine equivalent (mg): *M* (*SD*)	706.20 (323.36)	622.00 (294.16)	-	0.92 ^d^
Duration of illness: *M* (*SD*)	17.73 (5.80)	13.60 (4.96)	-	−2.38 ^e^
Exacerbation: *M* (*SD*)	5.80 (2.54)	6.23 (4.31)	-	−0.33 ^e^
Treatment interruptions: *M* (*SD*)	0.40 (0.57)	1.08 (1.50)	-	−1.58 ^e^
Global functioning in GAF: *M* (*SD*)	55.33 (13.70)	59.70 (13.82)	-	−1.05 ^d^

GAF = Global Assessment of Functioning; ^a^ One-way analysis of variance *F* test. ^b^ Kruskal–Wallis *H* test. ^c^ Chi-squared test. ^d^ Student’s *t* test. ^e^ Mann–Whitney *U* test.

**Table 3:** Relationship between DTI measures in the corpus callosum (when there were differences to HC), and psychopathological symptoms in both clinical groups.

Variable	Positive Symptoms in PANSS (*r*/*rho*)	Negative Symptoms in PANSS (*r*/*rho*)	Disorganization in PANSS (*r*/*rho*)	Affect in PANSS (*r*/*rho*)	Resistance in PANSS (*r*/*rho*)
Patients with deficit schizophrenia (DS)					
(*n* = 15)					
Fractional anisotropy (FA)					
Region 5	0.17 ^a^	0.16 ^a^	0.02 ^a^	0.18 ^a^	−0.10 ^a^
Mean diffusivity (MD)					
Region 5	0.01 ^a^	−0.16 ^a^	−0.08 ^a^	−0.34 ^a^	−0.13 ^a^
Patients with non-deficit schizophrenia (NDS)					
(*n* = 40)					
Mean diffusivity (MD)					
Region 4	0.09 ^b^	−0.17 ^b^	0.28 ^b^	−0.17 ^b^	0.32 ^b^

PANSS = Positive and Negative Syndrome Scale; ^a^ Pearson’s *r* correlation coefficient; ^b^ Spearman’s *rho* correlation coefficient.

## Data Availability

Data and materials for the experiments reported here are available from the corresponding author on reasonable request.

## Abbreviations

ANCOVA	Analysis of covariance
ANOVA	Analysis of variance
BNSS	Brief Negative Symptom Scale
CC	Corpus callosum
DS	Deficit syndrome
DSM-IV	Diagnostic and Statistical Manual of Mental Disorders Fourth Edition
DTI	Diffusion tensor imaging
FA	Fractional anisotropy
GAF	Global Assessment of Functioning
HC	Healthy controls
ICD-10	International Statistical Classification of Diseases and Related Health Problems Tenth Edition
M	Means
MD	Mean diffusivity
MINI	Mini-International Neuropsychiatric Interview
MRI	Magnetic resonance imaging
NDS	Non-deficit schizophrenia
PANSS	Positive and Negative Syndrome Scale
ROI	Region of interest
SD	Standard deviations
SNS	Self-evaluation of Negative Symptoms
TRS	Treatment-resistant schizophrenia
WM	White matter
